# Neuroimaging in the Epileptic Baboon

**DOI:** 10.3389/fvets.2022.908801

**Published:** 2022-07-14

**Authors:** C. Akos Szabo, Felipe S. Salinas

**Affiliations:** ^1^Department of Neurology, University of Texas Health San Antonio, San Antonio, TX, United States; ^2^Research Imaging Institute, University of Texas Health San Antonio, San Antonio, TX, United States; ^3^Department of Radiology, University of Texas Health San Antonio, San Antonio, TX, United States

**Keywords:** neuroimaging, genetic generalized epilepsy, baboon, PET, MRI

## Abstract

Characterization of baboon model of genetic generalized epilepsy (GGE) is driven both electroclinically and by successful adoption of neuroimaging platforms, such as magnetic resonance imaging (MRI) and positron emission tomography (PET). Based upon its phylogenetic proximity and similar brain anatomy to humans, the epileptic baboon provides an excellent translational model. Its relatively large brain size compared to smaller nonhuman primates or rodents, a gyrencephalic structure compared to lissencephalic organization of rodent brains, and the availability of a large pedigreed colony allows exploration of neuroimaging markers of diseases. Similar to human idiopathic generalized epilepsy (IGE), structural imaging in the baboon is usually normal in individual subjects, but gray matter volume/concentration (GMV/GMC) changes are reported by statistical parametric mapping (SPM) analyses. Functional neuroimaging has been effective for mapping the photoepileptic responses, the epileptic network, altered functional connectivity of physiological networks, and the effects of anti-seizure therapies. This review will provide insights into our current understanding the baboon model of GGE through functional and structural imaging.

## Introduction

Epilepsy is a condition of recurrent seizures or a single seizure in the setting of an epileptogenic lesions on a brain magnetic resonance imaging (MRI) scan or epileptiform abnormalities on a scalp electroencephalography (EEG) (1). Clinically, seizures are characterized by stereotyped, episodic changes in behavior. As seizures are rarely recorded in brief scalp EEG samples, clinicians rely on the detection of interictal (between seizures) epileptic discharges (IEDs), which can serve as markers for the seizure type. Epilepsies are classified mainly as focal, i.e., seizures beginning in one region of the brain, or generalized, i.e., seizures are associated with a simultaneous activation of both cerebral hemispheres (2). Developments in structural neuroimaging, intracranial EEG recordings, and analysis of histopathological samples collected by resective surgery in people with medically refractory epilepsy have provided important insights into the pathophysiology underlying focal epilepsies. Knowledge with regards to the mechanisms underlying generalized epilepsies (IGEs), are less apparent; they are mainly idiopathic (without a known etiology), not associated with neuroimaging abnormalities, and not amenable for intracranial EEG sampling or resective surgeries. While IGEs are presumed to be genetic in etiology, monogenetic mutations, mainly affecting ion channels, are encountered in only 3% of the cases (2). There is still a large gap for understanding likely polygenic affects underlying epileptogenesis in idiopathic (IGE) or genetic (GGE) generalized epilepsies, and animal models could provide new revelations with respect genetic and neurodevelopmental mechanisms.

Over fifty years ago, a prominent French neuroscientist, Robert Naquet, and his associates, Eva and Keith Killam et al. (3) first published their observations of photosensitivity, i.e., the predisposition of visual stimuli, such as flickering lights, to induce seizures. Photosensitivity of the baboon was quickly embraced for activation of seizures in a laboratory setting, allowing the recording of IEDs and seizures with scalp and intracranial EEG electrodes (4–6). The Senegalese *Papio hamadryas papio* (*P.h. papio*) appeared to be more photosensitive than other subspecies, including *P.h. anubis and cynocephalus* (7), remaining the preferred subspecies for electrophysiological evaluation and for testing the efficacy of known and novel anti-seizure medications. Photosensitivity was observed to be maximal in the morning hours and at intermittent photic stimulation (IPS) frequencies of 20–25 Hz. This model of photosensitivity was later adopted for testing known and novel anti-seizure medications (8, 9). However, technological improvements allowed electrophysiological investigations and anti-seizure medication testing in rodents and mice, decreasing interest for further developing the baboon model due to limitations related to cost and availability. Still, even after years of developing the baboon model, there is limited information regarding the underlying pathomechanisms and natural history.

Observation of spontaneous generalized tonic-clonic seizures (GTCS) in the largest captive baboon pedigree in the world, housed in the Southwest National Primate Research Center (SNPRC, Texas Biomedical Research Institute, San Antonio, Texas), reinvigorated research into this model. The pedigreed colony has 16,000 members over 6–8 generations, consisting mainly of *P.h. anubis, cynocephalus* and their hybrids; in contrast to the baboons studied in France, the founding members of this breeding colony originated from East Africa (10). On one hand, the pedigree presents a unique resource for evaluating the potential genetic effects of the epilepsy and led to the characterization of the epileptic phenotype. On the other hand, improved neuroimaging capabilities offers a new approach to evaluating underlying pathophysiology associated with this phenotype.

A retrospective case-detection survey of veterinary records between 1980 and 2007, demonstrated a prevalence of 26% for the expression GTCS or seizure-like behaviors in the pedigree (11). Forty-six spontaneous GTCS were recently semiologically characterized in 7 baboons; most of the seizures occurred in sleep or upon awakening (*12*). Preconvulsive semiologies were noted in 4 baboons, consisting of unilateral or bilateral rotatory behaviors and generalized or lateralized (myo)clonic activity; the ensuing convulsive portion of the seizures had a mean duration of 47 (+/-21) s. The total seizure duration was 54 (+/-21) s. Postictally, most of the baboons demonstrated myoclonus as they were recovering their upright posture following seizures (12). In addition to GTCS, generalized myoclonic, especially eyelid myoclonus, and absence seizures were recorded by on scalp video-EEG studies in 671 baboons (13). Generalized spike-and-wave discharges were noted in 324 (49%) baboons, and these were more commonly 4–6 Hz, but at times 2–3 Hz, frequency. Photoepileptic responses were recorded in 156 (23%) of the epileptic baboons. While these numbers may have been partially inflated with the use of low-dose ketamine (5–6 mg/kg) for sedation during scalp EEG studies; low-dose ketamine also activates generalized IEDs in asymptomatic animals predisposed to epilepsy. Nonetheless, these studies confirmed a genetic predisposition for epilepsy in this pedigree, and an electroclinical model that resembled juvenile myoclonic epilepsy (JME).

Only recently was a genetic etiology confirmed. Based upon whole genome sequencing in electroclinically well-characterized 42 epileptic and 19 controls, the RBFOX1 emerged as the only statistically significant association (14). RBFOX1 mutations have been identified in genetic focal and generalized epilepsies in humans and may act as a susceptibility gene in both humans and baboons (15, 16). RBFOX1 is an RNA-binding protein that regulates splicing of epilepsy candidate genes (e.g., *GABRG2, SYN1, KCNQ2, SCN8A, SLC12A5*) and plays a key role in neuronal excitation and may cerebral cortex development (17), changes to transcriptomic expression and splicing patterns of neuronal genes (18), and miRNA crosstalk that impacts homeostatic downscaling of excitatory synapses (19). As these gene interactions may be clinically relevant, larger samples of baboons will need to be studied in this pedigree. Furthermore, based upon a relatively consistent electroclinical phenotype within the pedigree, the epileptic baboon provides a suitable animal model for neuroimaging and evaluation of anti-seizure therapies.

This review will focus on the status of neuroimaging in the epileptic baboon. Based upon its phylogenetic proximity and similar brain anatomy to humans, the epileptic baboon provides an excellent translational model. Its relatively large brain size compared to smaller nonhuman primates or rodents, gyrencephalic structure compared to the lissencephalic brains of rodents and mice, and less variability in the cortical structures than humans, all contribute to the potential for identifying imaging biomarkers even in smaller cohorts than required for human studies. However, the studies described in this review were all performed in a single center, namely the SNPRC, and have not been validated by other centers. Nonetheless, because of the baboon's potential role in the neuroimaging of neurological disorders and treatment effects, several centers have developed PET and MRI templates. We will review the contribution of structural MRI as well as functional MRI and PET studies to our understanding of the baboon model, and more specifically the insights offered by functional imaging into the electrophysiological networks underlying photosensitivity, the epileptic network, and the effects of therapeutic interventions. We will also address gaps in the understanding of the structural and functional mechanisms, which will need to be addressed by prospective studies utilizing newly developed and/or complementary imaging techniques.

## Present State of Neuroimaging in the Epileptic Baboon

### PET and MRI Brain Templates

While the advantages of neuroimaging the epileptic baboons are numerous (described above), there are also many challenges. Some disadvantages to humans include the need to sedate or anesthetize baboons due to their size and strength, interference by the large snout and air-filled sinuses which can distort the acquisitions, and their smaller brain size. Nonetheless, due to the potential for translatability, several centers have developed neuroimaging platforms and brain atlases to facilitate neuroanatomical analyses.

These centers all strived to develop high-resolution neuroanatomical atlases to allow the identification of regions of interest and cortical/subcortical landmarks, based upon anatomical or MR templates that be co-registered with PET studies, and normalized to a standard space for whole brain analyses and statistical parametric mapping. Early templates were limited to axial sampling, using the anatomical segmentation of a single baboon brain, and were not electronically accessible. Riche et al. (20) produced an anatomical atlas cut into 15 mm thick slices in the orbitomeatal plane to simulate the transaxial PET acquisition and planar resolution. Subsequently, radioligand PET to identify and validate anatomically defined cortical or subcortical structures. Black et al. (21) published the first electronically accessible baboon template based upon the Davis and Huffman anatomical atlas from a single baboon and limited to the subcortical structures (22). Greer et al. (23) developed an MR-based high-resolution atlas (centered at the mid-sagittal line, AC-PC orientation), averaging 6 datasets that were reformatted using a voxel size of 0.5 mm^3^ to create representative MRI, that could be converted into PET space. A subsequent study aligned histological slices of baboon brain with anatomical MRI, first in two dimensions using block-face photographs of the brain slices, subsequently co-registering them in three dimensions with an *in-vivo* acquired MRI (24); this approach could link research relying on post-mortem microscopic material analysis with applications using *in-vivo* macroscopic imaging analysis. Further refinement of MRI templates was achieved by Love et al. (25), who created a template from 89 baboon brains of a heterogenous group of baboons based upon subspecies and sex to better represent their variability in brain morphometry. This template aimed to represent both hemispheres symmetrically to analyze side-to-side structural or functional differences and provided the first tissue probability maps, facilitating brain normalization or segmentation. Agaronyan et al. (26) created a fully segmented brain atlas, part semiautomatically, part manually, on an upscaled dataset using inter-slice interpolation. This atlas utilizes the earlier versions to guide segmentation, labeling and identification, hence achieving a higher resolution template to improve throughput analyses.

### Structural MRI

While structural MRI scans are essentially normal in people with IGE, statistical parametric mapping first demonstrated increased regional gray matter concentrations (GMC) or volumes (GMV) in the frontoparietal cortices, but their cortical distribution varied among studies (27–29). While histopathological substrate underlying increases of GMV or GMC is unknown, but it is thought to be related to cortical developmental effects or increased synaptic connectivity, potentially due to increased synaptic density and/or anomalous synaptic pruning in adolescence (30). Decreases in GMV have been also reported, most consistently affecting the thalami bilaterally (31, 32), suggesting chronic, seizure-induced damage.

**Figure 1:**
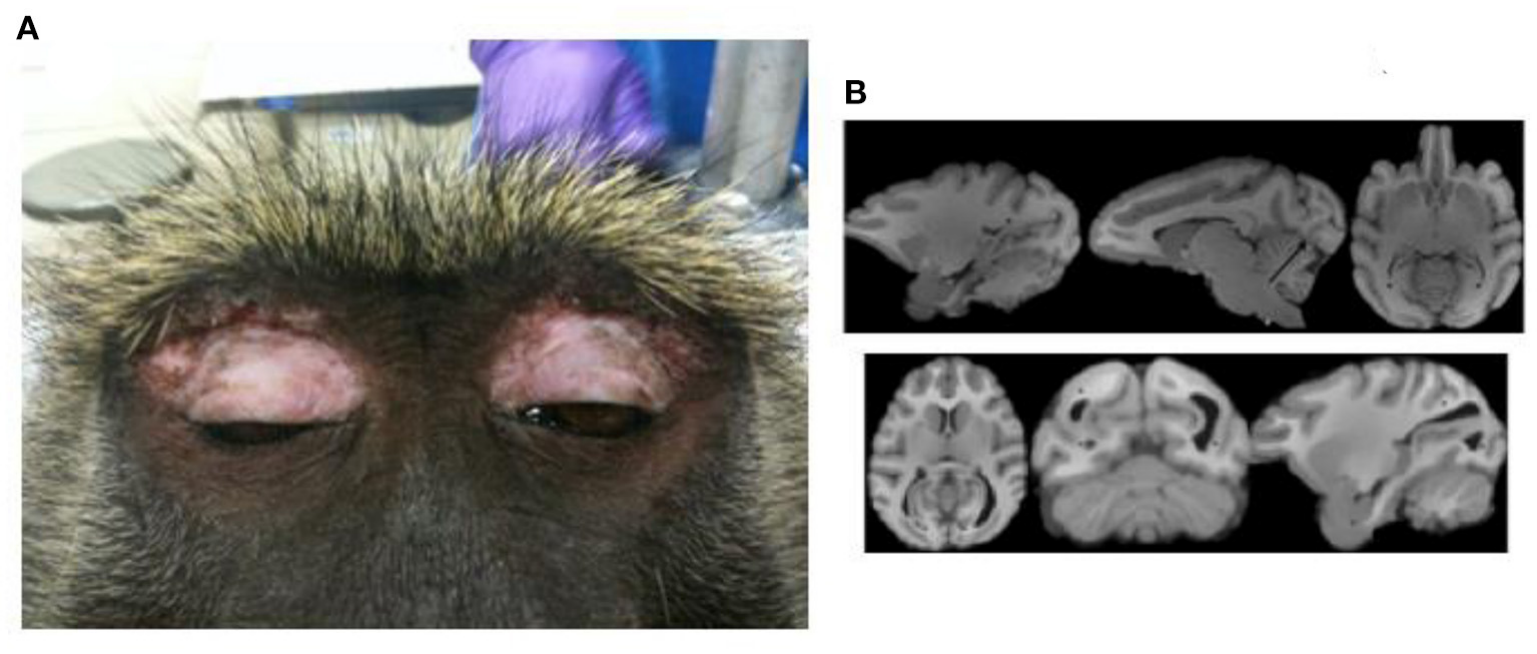
Craniofacial trauma and colpocephaly. **(A)** Demonstrates periorbital scarring due to repeated craniofacial trauma due to seizures. **(B)** Shows normal ventricular configuration (upper series) and enlarged occipital horns (lower series). Adapted from *Comparative Medicine*, references (33, 34), respectively.

The potential for correlating structural MRI findings with histopathology could allow the baboon model to provide insight into potential underlying neurodevelopmental abnormalities; however, until now histopathological studies have not demonstrated evidence of abnormal cortical development or dysplasia (33). The only common structural abnormalities are deviations in ventricular sized or shape. Occipital horn variants, including unilateral or bilateral elongation or enlargement (colpocephaly), occurred in 25% of MRI scans (T1-weighted three-dimensionally acquired) in 208 baboons (Figure 1B). Historically, colpocephaly is associated with craniofacial trauma, which are typically due to convulsive seizures leading to falls from elevations [Figure 1A; (34)]. Temporal horn enlargement is less common and lacks any association with trauma or epilepsy.

**Figure 2:**
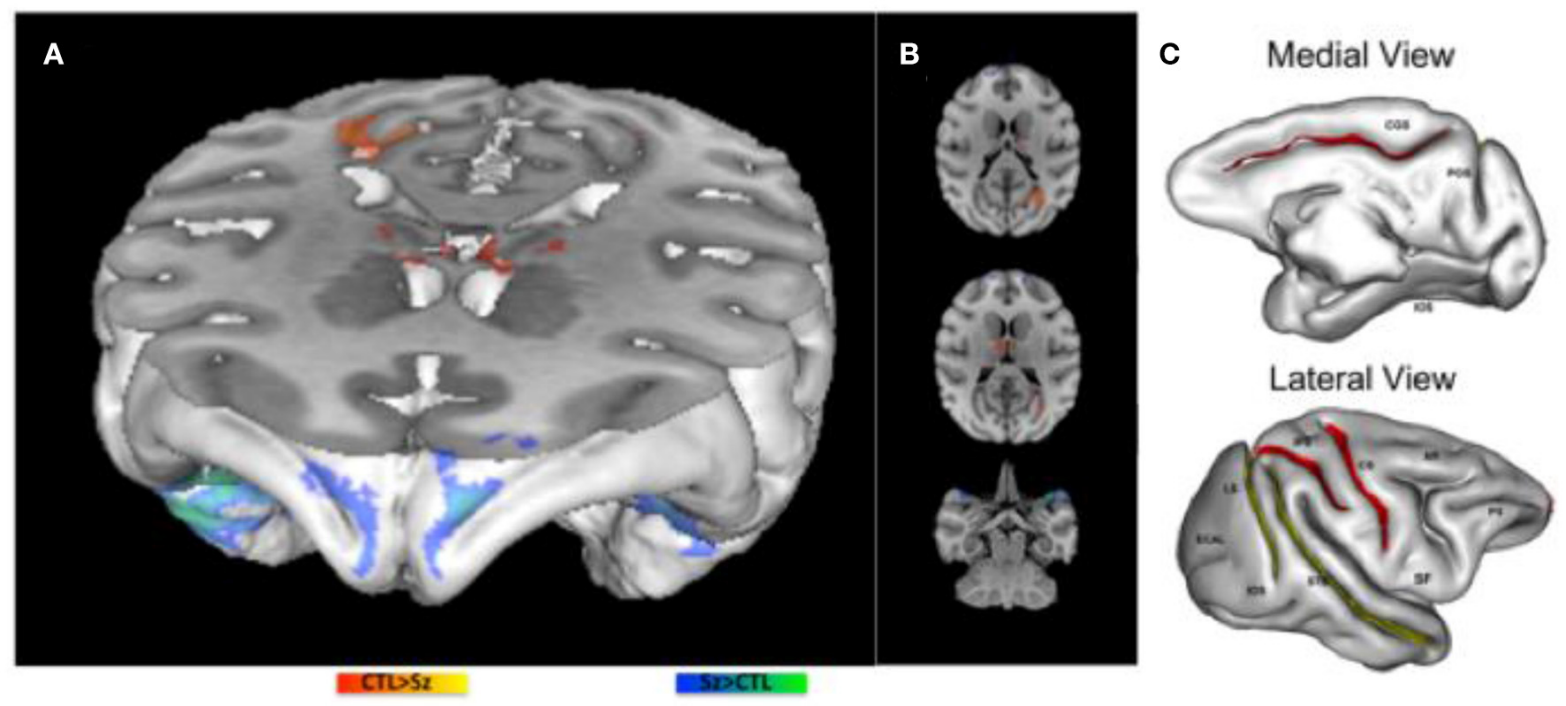
Morphometric MRI Analyses in the Epileptic Baboon. **(A,B)** demonstrate increases (blue) and decreases (red) in gray matter concentration in epileptic baboons compared to controls. **(C)** Demonstrate significant decreases (red) and marginally significant decreases (yellow) in sulcal areas of baboons with IEDs on scalp EEG compared to healthy controls [adapted from *Epilepsy Research*, references (35, 38), respectively].

Gyral and sulcal changes were compared between baboons with generalized interictal epileptic discharges (IEDs) on scalp EEG, some with witnessed seizures, others without, to those with normal scalp EEG and without witnessed seizures, using voxel-based morphometry [VBM, (35)]. Male brain volumes were ~15% larger than those of females. While no significant differences were noted between the IED+ and IED- groups, there was a subtle increase in brain volume in the baboons with IEDs. This finding was unexpected, but an increased thickness of the cerebral cortices, especially of the postcentral gyrus and associated parietal regions responsible for generating absence seizures, were also noted in the GAERS model (36). However, post-hoc analyses demonstrated significant decreases in sulcal areas involving the central, intraparietal and cingulate sulci [Figure 2C; (35)], all of which are associated with brain regions giving rise to generalized ictal and interictal discharges in rodent and humans IGEs (36, 37), suggesting either a neurodevelopmental abnormality or seizure-induced changes.

GMC changes were also documented with VBM in epileptic baboons [Figures 2A,B; (38)]. GMC increases were noted in the frontopolar, orbitofrontal and anterolateral temporal cortices bilaterally. Decreases of GMC were noted in the primary visual cortices and thalamic nuclei, including reticular, anterior and medial dorsal nuclei. Increased GMC may reflect an underlying neurodevelopmental abnormality, either due increased cellularity or the absence of synaptic pruning (30); in adolescents with new onset JME, increased GMV is noted more diffusely, and decreases over time to a lesser extent than in age-matched healthy controls. As these baboons were mainly adults, it is possible that GMC increases were also more diffuse early on, but then normalized either as a function of cortical maturation or due to seizure-induced cell loss (38). The latter explanation is suggested by histopathological exams of two adult epileptic baboon brains, demonstrating evidence of decreased cortical cellularity, especially of neurons, in the frontoparietal cortices, though maximal in the sensorimotor region, while frontopolar, orbitofrontal and visual cortices were relatively spared (39). On the other hand, decreased GMC was encountered in the primary visual cortices of epileptic adult baboons (38), a brain region that is implicated in photosensitivity and participating as an integral part the baboon's epileptic network (40). Consistent with human studies (31, 32), thalamic GMC was also reduced in the baboons, and involve specifically the reticular, anterior and medial dorsal nuclei of the thalamus, all of which are involved in the generation of generalized spike-and-wave discharges (38). To determine whether the sulcal morphometric and GMC/GMV abnormalities are neurodevelopmental in etiology and/or evolve before and during epilepsy requires prospective, serial neuroimaging in larger samples of young epileptic baboons, ideally starting before the onset of epilepsy.

Microstructural differences had not been published in baboons so far, but could play an important role in determining connectivity differences that may pre-exist the onset of epilepsy and those that result from seizure-related injury. Fractional anisotropy (FA) appeared to be increased sensorimotor and premotor pathways in people with IGE, but decreased in the internal and external capsules, in one recent seed-based study human IGE, suggesting seizure related plasticity (41). Increased motor connectivity is also apparent in people with JME and their asymptomatic siblings, supporting genetic predisposition to connectivity (42, 43). Similar to human IGE, seed-based analyses in the genetic absence epilepsy in rats from Strasbourg (GAERS) model also showed decreases in FA in the corpus callosum and internal capsule, but researchers were also able to demonstrate correlation of FA changes with seizure activity (44). Furthermore, treatment with ethosuximide appeared to prevent microstructural damage in these pathways and/or restore connectivity (44). These comparisons across species demonstrate the unique ability of animal models to illuminate mechanisms and treatment effects. Reanalyzing the baboon imaging data in a larger and better selected cohort may help validate these findings.

### Functional PET and MRI

#### Anesthesia

The advantage of performing functional PET or MRI studies in treatement-naïve baboons is counterbalanced by the need for sedation and anesthesia. Gas inhalant anesthesia using 1% isoflurane is particularly challenging, as the blood oxygen level dependent (BOLD) signal responses are progressively attenuated above 1.0 MAC. Ketamine, used at lower doses for sedation of baboons during scalp EEG recordings, does not attenuate BOLD or EEG signal and increases cerebral blood flow [CBF, (45)]. With prior EEG recordings used as a screening tool, an ideal infusion rate could be identified which would avoid activation of seizures as well as EEG suppression, usually between 4-6 mg/kg/hr, for functional imaging studies (46).

#### Photosensitivity

${\text{H}}_{2}^{15}$O-PET provides an excellent, minimally invasive method for studying cerebral blood flow (CBF) changes in association with visual stimulation in normal individuals (47–50). Similar to BOLD-fMRI, ${\text{H}}_{2}^{15}$O-PET takes advantage of neurovascular coupling to deliver visuospatial representations of brief electrophysiological events; the metabolic demand of activated or discharging neurons leads to localized changes in CBF and cerebral blood volume (47). ${\text{H}}_{2}^{15}$O-PET provides a more direct measure of CBF than BOLD-fMRI which relies on deoxygenation of venous blood but does not have the same spatial or temporal resolution of BOLD-fMRI.

${\text{H}}_{2}^{15}$O-PET studies were performed in the morning, when the baboons are maximally photosensitive, utilizing IPS at 25 Hz, the frequency range most likely to activate photoepileptic responses (3–5). Six to eight injections are performed, about 10–12 min apart as the tracer's half-life is only 2 min in duration. During resting scans, CBF changes are recorded for 90-second radiotracer uptake period. For activation scans, IPS was typically started one minute prior to ${\text{H}}_{2}^{15}$O-injection, as occipital CBF reaches a steady state after one minute of continuous stimulation (51). The averaged resting scans are subtracted from activation scans, and these PET images are either co-registered with the baboon's own MRI, or with an average MRI for group analyses (46).

**Figure 3:**
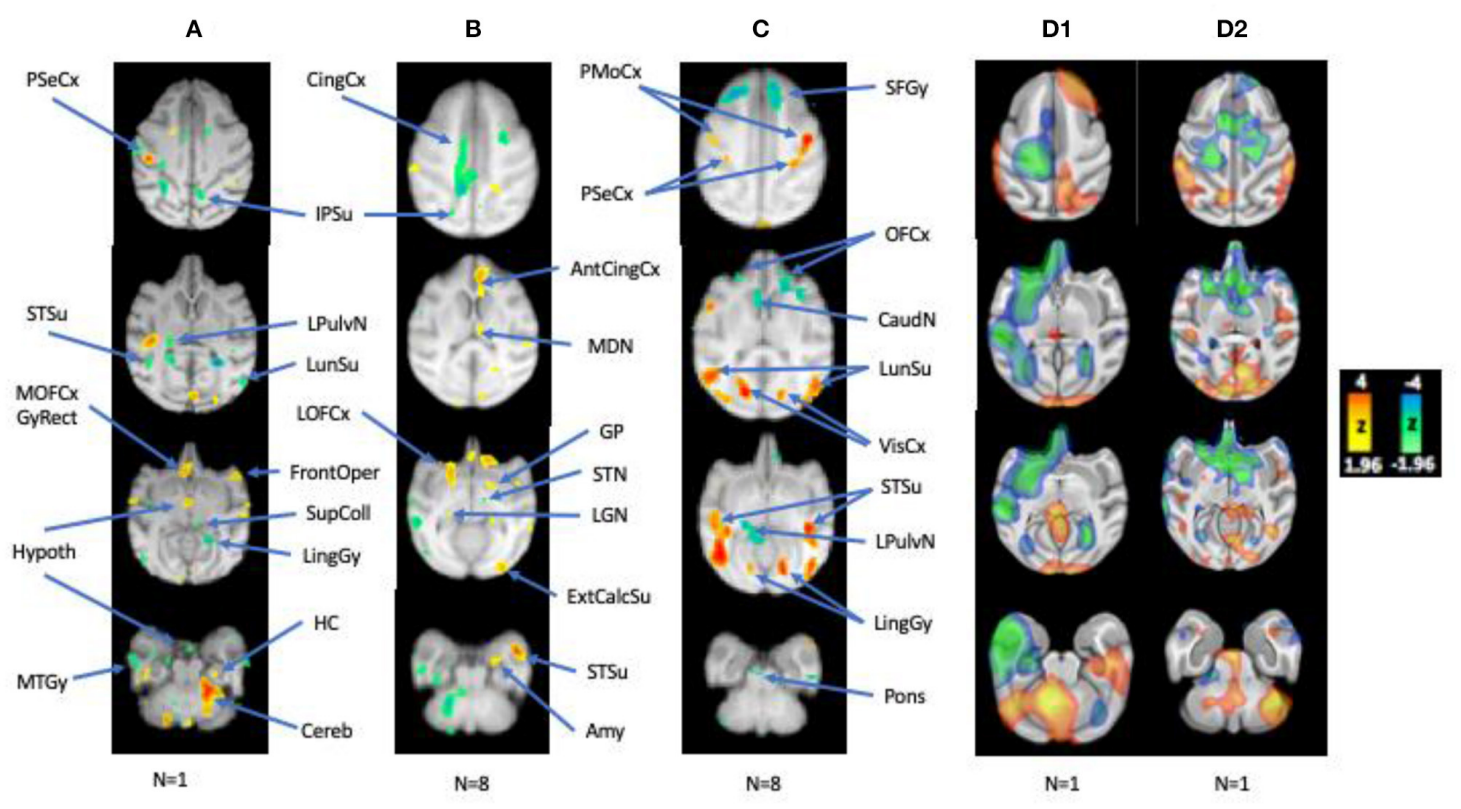
Regional CBF Changes with IPS, Correlated with IEDs, and during HF-VNS therapy. **(A,B)** Early and late CBF changes with IPS, **(C)** CBF changes correlated with interictal epileptic discharges, **(D1,D2)** CBF changes in a photosensitive baboon and a non-photosensitive animal related to HF microburst VNS Therapy at rest (compared to the resting-state average from nine epileptic baboons). L hemisphere is on the left. Adapted from references (46, 52, 53).

The photosensitive group exhibited more diffuse frontotemporal cortical and subcortical activations than control group (Figure 3B). Symmetrical cortical activations were mainly noted in the orbitofrontal cortices and anterior cingulate gyri, as well as the medial and anterolateral temporal cortices. Other activations were less symmetric, initially with a predominantly left hemispheric lateralization of the parietal and sensorimotor cortices when sampling CBF changes at IPS onset (Figure 3A), but shifting more to the right hemisphere once achieving steady state (Figure 3B). Activations were noted in both conditions in the right medial frontal regions including right anterior cingulate and orbitofrontal cortices, as well as the right mesiodorsal nucleus, though more bilaterally over time, reflecting effects of early and sustained activation of generalized IEDs recorded by scalp EEG. Similarly, the left posterior cingulate was also suppressed in both states, a brain region which is activated in the controls during IPS. There was diffuse deactivation of the basal ganglia and cortices early on, where deactivations in steady state pertained mainly to the caudal brainstem and cerebellum. Also subcortically, activations were noted in the left putamen, compared to the right putamen in controls. Another unexpected finding was the absence of occipital lobe activation in the photosensitive baboons; in photosensitive humans, EEG-fMRI demonstrates consistent occipital activation at the onset of IPS (54). While there was diffuse occipitoparietal activation noted in the control baboons, photosensitive baboons only showed minimal occipital activation early on during IPS, suggesting the possibility of an endogenous cortical inhibition induced by IPS. Nigral inhibitory pathways were also activated, reflected by regional CBF increases noted in the midbrain and globus pallidum, in baboons exhibiting myoclonic seizures during the tracer uptake period compared to baboons with only IEDs (55). This appears to be an important inhibitory pathway; even structural MRI demonstrated enlargement of the globus pallidum in epileptic baboons compared to controls (38).

#### The Epileptic Network

With the help of EEG-fMRI, the epileptic network underlying absence seizures has been well-defined in humans and rodents (44, 56). The onset and resolution of paroxysmal generalized spike-and-wave complexes in people with absence and myoclonic seizures are associated with transient activation of the medial frontothalamic network (37, 56). BOLD changes precede the paroxysmal EEG discharge by about 15 s, with initial rises in BOLD signal occipitally, parietally and orbitofrontally (56, 57); nonetheless, the order latency of activations and deactivations can vary widely between subjects (57). The generalized spike-and-wave is followed by sustained decreases in BOLD signal across the frontoparietal cortices. This is one reason for overall decreases in frontoparietal BOLD signal during random sampling in IGE patients.

But even without EEG-fMRI, functional PET was able to demonstrate activation of the corticothalamic network during IPS (46). Furthermore, correlation analyses of CBF changes with resting IED rates also resulted in the activation of the posterior aspects epileptic network (Figure 3C; 56), which included symmetrical activations of the primary visual cortices, occipito- and temporo-parietal association cortices, insula, and sensorimotor cortices, all brain region that were active both ictally and interictally during intracranial EEG recordings (40). However, other brain regions that were activated by IPS and were electrophysiologically active on EEG, namely the anterior cingulate, orbitofrontal cortices, even the thalamus, were either deactivated or not activated. As suggested by EEG-fMRI recordings of absence seizures in humans, near-infrared recordings of oxyhemoglobin, deoxyhemoglobin and total hemoglobin in the GAERS cortex, deoxygenation and CBV are decreased before the onset and after the end of the generalized spike-and-wave discharge (58), which may account for the paradoxical deactivation is these brain regions. Subcortical CBF changes were mainly represented by deactivations in the left caudate and lateral pulvinar nucleus, which were activated during IPS, in the pontine nucle and cerebellum.

**Figure 4:**
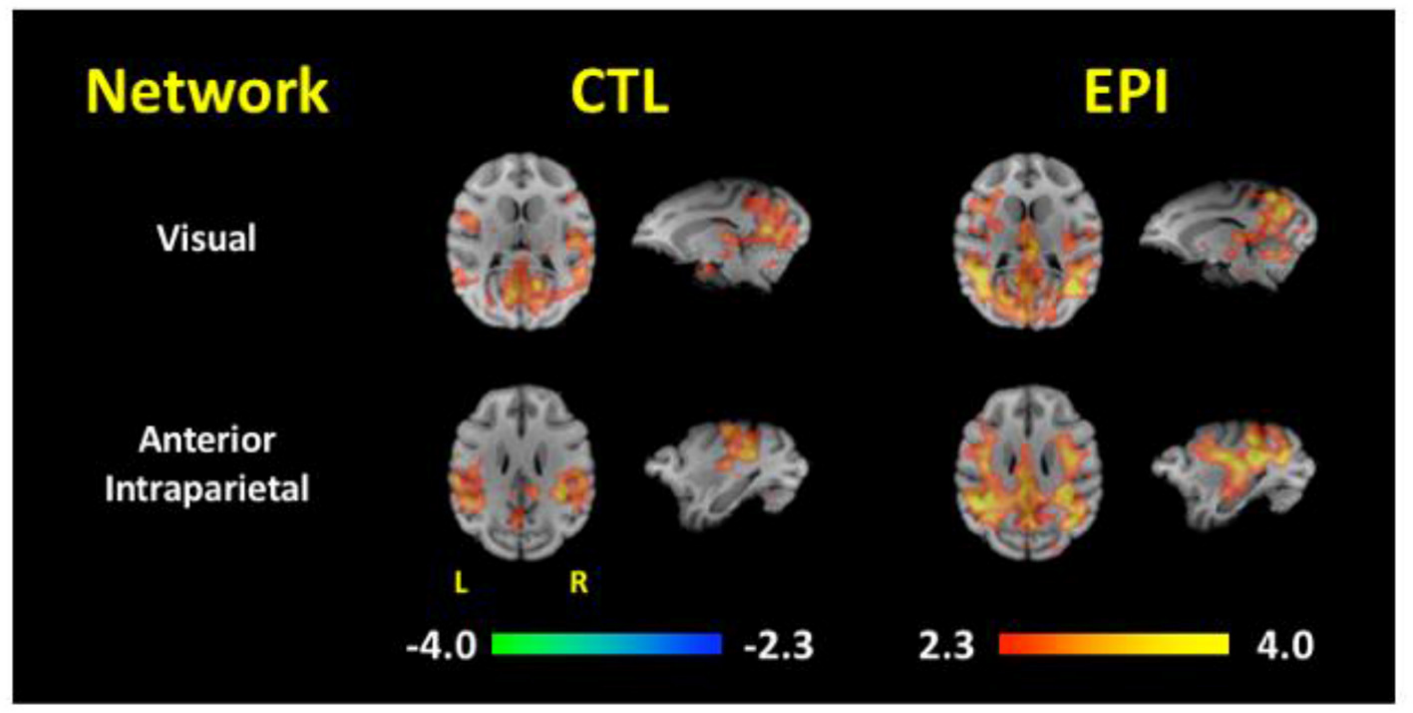
Functional Connectivity Maps for the Visual and Anterior Parietal Networks. Functional connectivity maps are compared between asymptomatic healthy control (CTL) and epileptic (EPI) baboons. Note the increased connectivity of both visual and anterior partial networks to the medial thalamus and frontocentral cortices [Adapted from *Epilepsia*, reference (59)].

This network was also supported by FC analyses comparing 10 epileptic and 10 control baboons, matched for gender, age, and weight (59). Independent component analyses (ICA) identified 14 unique components/networks, and FC maps were generated for each group's brain networks (Figure 4). The epileptic group demonstrated network-specific differences in FC when compared to the control animals: sensitivity and specificity of the two groups' functional connectivity maps differed significantly in the visual, motor, amygdalar, insular, and default mode networks. Significant increases of FC were found in visual cortices of the epilepsy group's maps for the default mode, cingulate, anterior parietal, motor, visual, amygdalar, and thalamic networks. More importantly, Figure 4 demonstrates that the connectivity maps of the visual and parietal networks reconstitute the entire epileptic network, including for the corticothalamic pathways that express generalized ictal and interictal epileptic discharges.

#### Treatment Effects

Functional neuroimaging can also be used to evaluate acute or chronic treatment effects for anti-seizure medications or devices. We compared FC changes after intravenous injection of valproic acid (VPA 20 mg/kg) and following 1-week of orally administered VPA (20–80 mg/kg/day) between epileptic and control baboons (60). Similar to study above (59), FC was increased in most cortical networks of the epilepsy group, but less so for the subcortical networks (60). After intravenous VPA, FC was increased for the basal ganglia network in the epileptic baboons with respect to the medial frontal cortices, but decreased for longer cortico-cortical pathways. Increased basal ganglia connectivity may reflect an acute upregulation of cortical inhibition by subcortical networks, while decreasing cortico-cortical connectivity is likely to reflect decreased activation of the larger epileptic network. Cortical and basal ganglia connectivity was also increased with pontine nuclei and cerebellum, supporting potential activation of another pathway modulating cortical excitability. After oral VPA therapy, FC in the epileptic baboons approached control values in the amygdalar, precuneus, left parieto-occipital, parietal, orbitofrontal, and secondary visual areas. Increases of FC were noted for the medial frontal/(peri)cingulate and pontine networks, which may reflect brainstem-mediated neuromodulation *via* activation of biogenic amine secretion and neurotransmission.

In a more recent study, ${\text{H}}_{2}^{15}$O-PET changes were compared between high-frequency (HF; 300 Hz) microburst, and standard, low-frequency (LF; 30 Hz) Vagal Nerve Stimulation (VNS) Therapy in two baboons with GGE, including one with photosensitivity (53). The baboons were selected based on video recordings and scalp EEG studies. Both were implanted with Sentiva™ M1000 devices capable of stimulating at LF- and HF-frequencies. CBF changes were compared for both modes of stimulation and resting scans in the first study. While spontaneous scalp IEDs were reduced in both baboons by HF- and LF-therapies, HF-VNS Therapy completely suppressed IEDs in one baboon (D2). Regional CBF changes were overall consistent between the two modes of therapy in both baboons with respect to the activation of the superior colliculus and cerebellum (Figures 3D1,D2). IED suppression by HF-VNS Therapy in one baboon was associated with bilateral deactivations of the frontal and temporal cortices, anterior cingulate and striatum, and of the medial thalamus, while the pons and cerebellum were both activated (Figure 3D2). Some therapeutic targets for both LF- and HF-VNS Therapy appeared to be subcortical, including the superior colliculus, brainstem nuclei, as well as the cerebellum, all structures that were either deactivated or inactive during IPS or spontaneous IEDs.

## Future Directions

Structural and functional neuroimaging has contributed to our understanding of brain networks and pathophysiology underlying GGEs in humans and baboons alike, and the potential for translation is extraordinary. As such, different modalities may be used to characterize brain developmental effects which may contribute to the expression of seizures as well as seizure-induced cortical and subcortical damage. Serial neuroimaging could lead to a new perspective on the natural history of GGE in the baboon and identify neuroimaging biomarkers for epileptogenesis and sudden unexpected death in epilepsy (SUDEP) (61). As demonstrated by the studies above, GMC/GMV measurements and FC connectivity changes could track the evolution of epileptic networks. Quantitative trait analyses could map genetic effects on brain development and connectivity (10, 62). Several newer PET-ligands could contribute to our understanding of neurodevelopmental mechanisms associated with epileptogenesis. As SV2A modulates the exocytosis of synaptic vesicles, SV2A-PET can quantify synapses and synaptic connectivity in the cerebral cortex (63, 64). Translocator protein or TSPO-PET, on the other hand, can evaluate inflammatory changes that may also contribute to epileptogenesis (65). Both can also be used to monitor seizure-related loss of cortical and brainstem neurons. Ultimately, the epileptic baboon model may be used to validate of neuroimaging markers by electrophysiological testing and the availability of brain tissue.

## Author Contributions

CS and FS designed the studies and performed the research and analysis. CS wrote the review. FS provided critical input. All authors contributed to the article and approved the submitted version.

## Funding

The study utilized the SNPRC grant P51 RR013986 through the NCRR by the Office of Research Infrastructure Programs, P51 OD011133, and was conducted in facilities constructed with support from Research Facilities Improvement Grants C06 RR013556, C06 RR014578, and C06 RR015456. This study was supported by the National Institutes of Health research grants 1 R01 NS047755 to Jeff T. Williams, NIBIB K01 EB006395 to Peter Kochunov, NINDS F32 NS066694 to FS, and SNPRC (NIH) P51 RR013986, NINDS R21 NS065431 and R21 NS084198 and by Livanova (London, UK) to CS. The funders of the research performed by the authors were not involved in the study design, data collection, interpretation of the data, the writing of this article or the decision to submit it for publication.

## Conflict of Interest

The authors declare that the research was conducted in the absence of any commercial or financial relationships that could be construed as a potential conflict of interest.

## Publisher's Note

All claims expressed in this article are solely those of the authors and do not necessarily represent those of their affiliated organizations, or those of the publisher, the editors and the reviewers. Any product that may be evaluated in this article, or claim that may be made by its manufacturer, is not guaranteed or endorsed by the publisher.

## Figures and Tables

**Table:** 

**Cortical regions of interest**	**Subcortical structures**
(M/L)OFCx, medial/lateral orbitofrontal cortex	CaudN, Caudate nucleus
FrontOper, frontal operculum	LPulN, lateral pulvinar nucleus
SFG, superior frontal gyrus	LGN, lateral geniculate nucleus
GyRect, gyrus rectus	SupColl, superior colliculus
PMoCx, primary motor cortex	Cereb, cerebellum
PSeCx, primary sensory cortex	GP, globus pallidum
VisCx, visual cortex	STN, subthalamic nucleus
LingGy, lingual gyrus	Hypoth, hypothalamus
(Ant)CingCx, (anterior) cingulate cortex	MDN, mediodorsal nucleus thalami
MTGy, middle temporal gyrus	HC, hippocampus
STSu, superior temporal sulcus	AMY, amygdala
ExtCalcSu, external calcarine sulcus	
IPSu, intraparietal sulcus	
LunSu, lunate sulcus	
